# Cerebral MRI and Clinical Findings in Children with *PTEN* Hamartoma Tumor Syndrome: Can Cerebral MRI Scan Help to Establish an Earlier Diagnosis of PHTS in Children?

**DOI:** 10.3390/cells9071668

**Published:** 2020-07-10

**Authors:** Michaela Plamper, Mark Born, Bettina Gohlke, Felix Schreiner, Sandra Schulte, Vera Splittstößer, Joachim Woelfle

**Affiliations:** 1 Children’s Hospital, Department of Pediatric Endocrinology and Diabetology, University of Bonn, Venusberg-Campus 1, 53127 Bonn, Germany; bettina.gohlke@ukbonn.de (G.B.); Felix.Schreiner@ukbonn.de (S.F.); Sandra.Schulte@ukbonn.de (S.S.); Vera.Splittstoesser@ukbonn.de (S.V.); joachim.woelfle@uk-erlangen.de (W.J.); 2 Department of Radiology, Pediatric Radiology, University Hospital Bonn, Venusberg-Campus 1, 53127 Bonn, Germany; mark.born@ukbonn.de; 3 Children’s Hospital, University of Erlangen, Loschgestrasse 115, 91054 Erlangen, Germany

**Keywords:** *PTEN*, PHTS, children, cMRI, enlarged perivascular spaces, Virchow–Robin spaces, white matter abnormalities, macrocephaly

## Abstract

Background: *PTEN* Hamartoma Tumor Syndrome (PHTS) is caused by germline autosomal-dominant mutations of the tumor suppressor gene *PTEN*. Subjects harbour an increased risk for tumor development, with thyroid carcinoma occurring in young children. Establishing a diagnosis is challenging, since not all children fulfill diagnostic criteria established for adults. Macrocephaly is a common feature in childhood, with cerebral MRI being part of its diagnostic workup. We asked whether distinct cMRI features might facilitate an earlier diagnosis. Methods: We retrospectively studied radiological and clinical data of pediatric patients who were presented in our hospital between 2013 and 2019 in whom *PTEN* gene mutations were identified. Results: We included 27 pediatric patients (18 male) in the analysis. All patients were macrocephalic. Of these, 19 patients had received at least one cMRI scan. In 18 subjects variations were detected: enlarged perivascular spaces (EPVS; in 18), white matter abnormalities (in seven) and less frequently additional pathologies. Intellectual ability was variable. Most patients exhibited developmental delay in motor skills, but normal intelligence. Conclusion: cMRI elucidates EPVS and white matter abnormalities in a high prevalence in children with PHTS and might therefore aid as a diagnostic feature to establish an earlier diagnosis of PHTS in childhood.

## 1. Introduction

*PTEN* hamartoma tumor syndrome (PHTS) is caused by germline autosomal-dominant mutations of the tumor suppressor gene *PTEN*. PHTS encompasses different syndromic disorders including Cowden syndrome (CS), Bannayan Riley Ruvalcaba Syndrome (BRRS), Lhermitte Duclos Syndrome, Proteus and Proteus like syndrome. In addition, juvenile polyposis of infancy, autism spectrum disorders with macrocephaly and children with macrocephaly and developmental delay and/or cognitive impairment are associated with *PTEN* gene mutations. All individuals with molecularly proven *PTEN* mutation are at increased risk of developing benign or malignant tumors and therefore benefit from cancer surveillance strategies. Most frequently, tumor development is described in breast, endometrium and thyroid. In addition, other organs like the skin, colorectal, renal and central nervous systems can be affected [1]. The development of thyroid carcinoma has been described in young children [2,3,4]. Thus, it is important to make an early diagnosis to implement screening examinations.

Macrocephaly (MC) is an important clinical feature to initiate genetic testing of the *PTEN* gene and therefore an important criterion for a diagnosis of *PTEN* gene mutation and PHTS in childhood [5,6]. Several publications reported a prevalence of up to 100% in pediatric patients [7,8,9,10,11]. In early childhood, macrocephaly seems to be the only or the most evident symptom. The diagnostic workup of non-familial increased head circumference in children (> + 2 SDS above the age and population related mean or >97th percentile) is done using a cerebral MRI scan.

In the current literature, different neuroimaging features associated with *PTEN* mutations have been reported, including cerebellar dysplastic gangliocytoma (Lhermitte–Duclos disease (LDD)), meningioma, vascular malformations [12], localized malformations such as focal cortical dysplasia, white matter abnormalities [13], dilated Virchow–Robin spaces [14] and polymicrogyria [15]. Since our institution hosts a rare disease center for familiar tumor syndromes, we have the opportunity to supervise a relatively large pediatric cohort of patients with PHTS. Most of these patients had received at least one cerebral MRI, frequently before establishing a diagnosis of PHTS. Based on the pre-diagnostic MRI findings, we wondered whether distinct features of cerebral MRIs in PHTS might help to establish an earlier diagnosis in children.

## 2. Methods

We retrospectively studied all available data of our cohort of patients with molecular-proven PHTS, who had presented in our pediatric department for endocrinology between the years 2013 and 2019.

The PHTS patients did not primarily visit our department because of endocrinological problems, but because we established a PHTS clinic as part of our center for rare diseases. Because all data presented in this analysis were included in routine care of these subjects and no additional examinations were performed, we did not request for additional ethics clearance.

Clinical features of our patients were collected, concentrating on psychomotor milestones of motor and language development as well as additional neurological findings. In patients in whom a cerebral MRI scan (cMRI) had been performed previously, we asked the families for permission to analyze their cMRI. All participating families gave consent for analysis of pre-diagnostic radiological data. Either families gave us their own copies of cMRI for analysis or they gave us consent to contact the operating institutes to send us a copy of the cMRI pictures.

The majority of MRI scans were performed as part of the initial clinical routine in a local institution, so that cMRI techniques did not follow a standardized approach.

The examinations were performed with scanners from different manufacturers, and with different examination protocols in local operating institutes. In all cases, a field strength of 1.5 T was used. Five patients had only native cMRI, all other patients had at least one MRI with contrast medium. In almost all examinations the following standard sequences were used: T2 weighted fast spin echo, T1 spin echo and fluid attenuated inversion recovery (FLAIR). On the other hand there were significant differences in the technical parameters, for example the slice thickness in fast spin echo varied from 2.5 to 6 mm and in T1 spin echo from 3 to 6 mm. Soft tissue contrast is significantly influenced by the echo time, which varied from 80 to 130 ms in fast spin echo, and from 90 to 150 ms in FLAIR. Inversion time in FLAIR differed from 1800 to 2850 ms. Because of the described diversity, a quantification of the different examinations and their signal intensity would be difficult. Moreover, signal intensity especially of the white matter is age-dependent. Therefore, we did not use a software program to quantify the signal intensity.

## 3. Results

We included 27 pediatric patients (18 male and nine female patients) in the present study. Age at diagnosis of the *PTEN* mutation ranged from eight months to 13.5 years.

In most cases, genetical analysis was performed within the diagnostic workup for a combination of macrocephaly, developmental delay and other clinical features such as penile freckling or lipoma. In two cases (patient 9 and 16), the responsible physicians recommended *PTEN* gene analysis because of the combination of macrocephaly and enlarged perivascular spaces (EPVS). In two additional patients, diagnosis was made, because of a thyroid pathology and macrocephaly (follicular adenoma and macrocephaly-patient 10; follicular carcinoma and macrocephaly-patient 19).

The youngest patient, who had received a cerebral MRI scan was three months old. The age distribution at performance of cerebral MRI scan is depicted in Table 1.

### 3.1. Macrocephaly

As reported before [11], all patients of this study exhibited macrocephaly by the age of two years. Stratified for different age groups the mean head-circumference SDSs for boys was above +4 SDS, and for girls, above +3 SDS.

### 3.2. MRI

Nineteen out of twenty-seven patients (13/18 male patients (72%) and 6/9 female (67%) patients) had received at least one cerebral MRI scan. The age at cMRI in our patient cohort ranged from infancy to early adolescence (for details see Table 1); different institutions used different technical conditions. Some patients received one or more follow up cerebral MRI examination to follow-up on conspicuous results.

**Figure 1:**
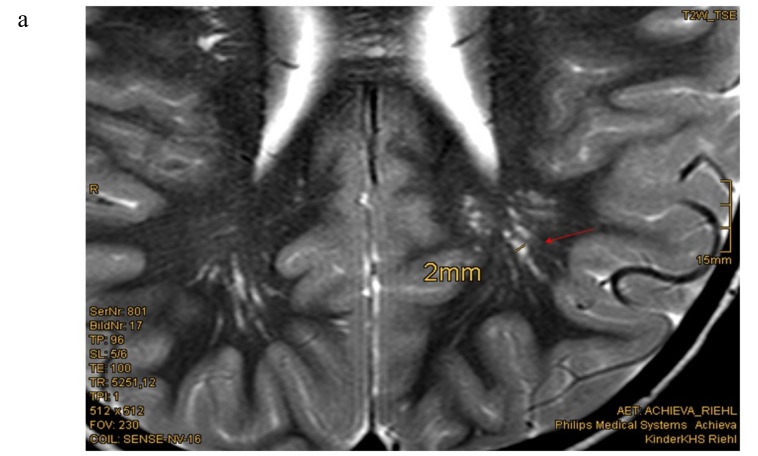

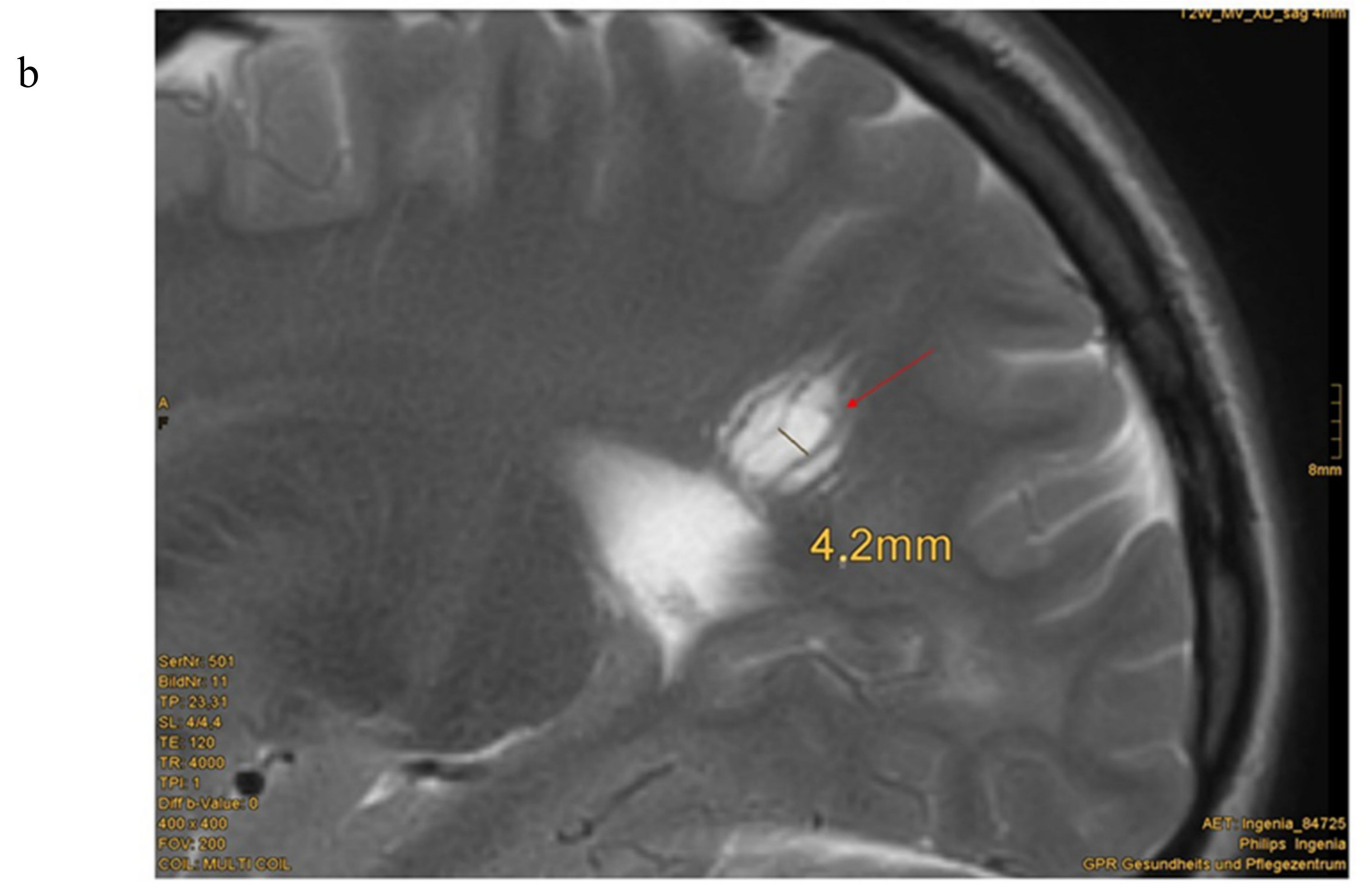
Enlarged perivascular spaces (EPVS) in cerebral MRI (**a**) Patient 15: 4.5 year-old boy, T2w-image, EPVS 2 mm diameter [Courtesy Kinderkrankenhaus Kliniken der Stadt Köln]. (**b**) Patient 23: 12.5 year old girl, T2 weighted image, sagittal view, pronounced EPVS up to 3 mm diameter [Courtesy Dr. A. Wieschen, Institut für Radiologie und Nuklearmedizin, GPR Klinikum Rüsselsheim].

**Figure 2:**
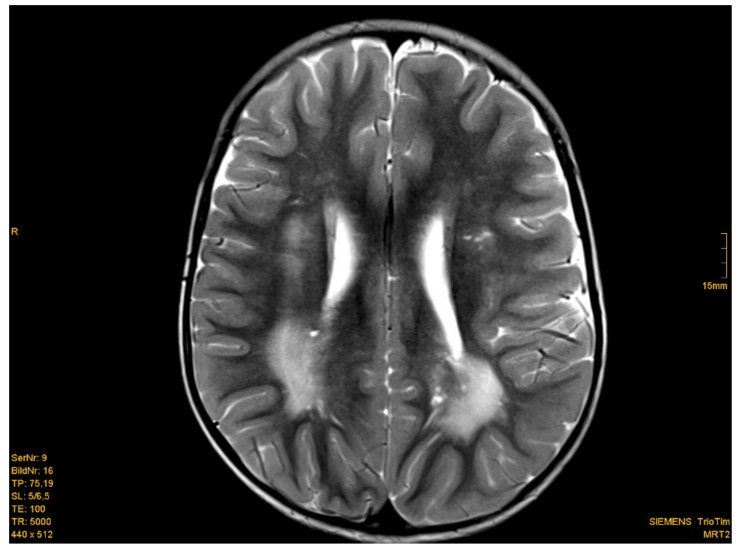
Example of white matter abnormalities. Patient 6: 2 year-old boy, T2 weighted image. Posteriorly increased signal intensity of the white matter. [Courtesy Dr. Scharper, Radiology University Düsseldorf].

Eighteen of nineteen patients exhibited variations in cMRI. In 17 of 19 patients, we were able to re-analyze the MRI scans. In two cases, we only obtained medical reports, which were reported to be normal, even though enlarged perivascular spaces were described in one of these two patients. Medical reports of our patient cohort described enlarged perivascular spaces (EPVS), also known as Virchow–Robin Spaces (VRS), in six patients. However, when we re-analyzed the MRI pictures with special focus on EPVS, we found enlarged perivascular spaces in a total of eighteen patients (18/19). In some patients, these findings were quite evident (Figure 1b). Seven patients (7/17) presented with white matter abnormalities (Figure 2). Further pathologies included a cavernoma at the right side of the cerebellum, subependymal heterotopia at the top of the lateral cerebral ventricle, arachnoid cysts (in two), a Chiari malformation type I and a clinical diagnosis of pseudotumor cerebri with ventriculoperitoneal shunt in one patient (Table 1).

### 3.3. Psychomotor Development

The clinical presentation of our patients was variable. Most infants exhibited a delay of early motor development. The age when patients started walking independently, as one of the important milestones of motor development, ranged from 14 to 30 months in male patients (mean 20 months, median 18 months) and from 13 to 29 months in female patients (mean 21.3, median 20 months).

In twelve patients the treating pediatric neurologists described muscle hypotonia to a variable degree. Most parents reported rapid fatigue when their children needed to walk a longer distance. Two patients needed medical devices (such as a wheelchair) to handle longer distances. Two boys and one girl were tested and diagnosed with an autism spectrum disorder, which was the dominant clinical problem of these patients. One of the boys was seriously affected and presenting with no expressive speech by the age of 12 years. The two other patients suffered from milder forms of autism spectrum disorders. The remaining patients had not been tested for autism spectrum disease. Some patients underwent developmental testing. However, since testing was part of the individual clinical routine preceding a diagnosis of PHTS, different test protocols were used, and patients were tested at different (mostly very young) ages. Therefore, it was not possible to compare the results of these tests systematically in this retrospective analysis. The majority of patients who had received IQ testing demonstrated an IQ within the normal range (IQ 85–115), but were reported to have difficulties in logical reasoning, attention and a slow processing speed. For further details of the tested patients (age, testing protocol and results), see Table 1. A greater number of our patients were not tested, because the patients showed no clinical hints for problems regarding their intellectual development. They visited regular schools with appropriate school performances, two had already graduated from secondary school and started university. Overall, intellectual ability was quite variable in this patient group. Behavioral problems were reported in a minor number of our patients, for example obsessive-compulsive disorder, attention deficit hyperactivity disorder (ADHD), autism or impulsivity. For further details see Table 1. Almost all patients were reported to show problems in motor coordination.

### 3.4. Additional Phenotypical Features

We have already published some of the additional phenotypical features (in particular auxology, thyroid pathology) of this cohort elsewhere [2,11].

Thyroid: In this current cohort, 14/27 patients showed a thyroid pathology. Half of them (7/14) had undergone thyroid surgery due to highly suspicious lesions. Histopathological findings included a papillary microcarcinoma in a six-year-old boy and a follicular carcinoma in a 13-year old girl, as well as nodular goiter and follicular adenoma. Other patients demonstrated signs of autoimmune thyroid disease and thyroid nodules or cysts.

Gastrointestinal findings: Colonoscopy had been performed in five patients so far. In four of five patients, multiple polyps of the gastrointestinal tract were found. One patient was a carrier of an additional *BMPR1A* deletion and thus had to undergo colectomy for disease control.

Skin: Special skin findings in this patient group were penile freckling (in 10 of 18 male patients; 56%), lipoma (15/27 patients; 56%), hemangioma (5 patients; 18.5%) and trichilemmoma in two patients.

## 4. Discussion

Diagnosis of PHTS in childhood can be challenging. First, PHTS is a rare disease with an estimated prevalence of 1:200,000 to 1:250,000 [16,17] and is therefore still not well known in the medical community. Second, children often do not fulfill the clinical criteria of Cowden Syndrome (CS), which has been established for adults with CS, with CS being the best known condition of the PHTS spectrum [18,19]. Pediatric clinical features for *PTEN* testing have been published by Tan et al. in 2011 [8] and are particularly known by specialists in this field, but a broader knowledge in the pediatric community is lacking. Most children and adolescents present clinical aspects of BRRS, which encompasses macrocephaly, hamartoma (for example lipoma, hemangioma and intestinal polyps) and penile freckling in male patients [5,6], whereas some children only exhibit macrocephaly with a variable degree of developmental delay or cognitive impairment. In the end, BRRS and CS can be seen as one condition with variable expression and age-related penetrance [20].

As mentioned above, macrocephaly is probably the most evident symptom of PHTS in childhood and cerebral MRI is one aspect of its diagnostic workup. In this context, it is useful to distinguish whether the child suffers from megalencephaly, defined by increased growth of cerebral structures, related to dysfunctional anomalies during brain development in the neuronal proliferation/migration or whether the increased head circumference is caused by macrocephaly, which is linked to various events including anomalies of bone, subdural fluid collections, arteriovenous malformations, hydrocephalus or others [21]. Strictly speaking, in most patients with PHTS we should speak of megalencephaly and not macrocephaly [21,22,23], but in most scientific papers these terms are used interchangeably [20].

As expected, the reason of the increased head circumference in this patient cohort was megalencephaly. In addition, we detected an unexpected high rate (18/19; 95%) of enlarged perivascular spaces (EPVS). Enlarged perivascular spaces or so-called Virchow–Robin Spaces (VRS) are perivascular extensions of the pia mater accompanying the perforating arteries of the brain [24]. They are filled with fluid and can be seen in people of any age, but their prevalence increases with advancing age [25,26]. It is not clear, whether they are merely a benign manifestation of ageing or whether EPVS are a form of relatively distinct pathological change seen in the ageing brain and in various disease states. One study found that EPVS were associated with cognitive decline in otherwise healthy elderly men [27].

A high percentage of EPVS in pediatric patients with *PTEN* mutation and macrocephaly has been described in a previous publication [28]. Vanderver and coauthors described enlarged perivascular spaces with or without periventricular white matter abnormalities as a relatively consistent imaging feature in 23 patients with proven *PTEN* mutation and macrocephaly.

They concluded that the presence of EPVS and/or white matter abnormalities in patients with macrocephaly and developmental delay or autism spectrum disorder should give reason to test for the *PTEN* gene mutation. Our results strongly underline this recommendation, since another frequent observation (37%) in almost half of our patients were white matter abnormalities. We included one patient with heterozygous deletion of the *PTEN* and *BMPR1A* genes who exhibited EPVS and white matter abnormalities. We cannot prove that MRI findings are related to either *PTEN* or *BMPR1A* mutation. However, to the best of our knowledge, EPVS as well as white matter abnormalities have only been described in patients with *PTEN* gene mutation, but not in patients with *BMPR1A* mutations. Whereas germline mutations in *PTEN* are associated with macrocephaly, with brain MRI showing markedly larger brain volumes as well as increased white matter hypointensities compared to other patients and healthy controls [28,29,30]. Patients with white matter abnormalities in our study exhibited a wide clinical spectrum from severe developmental delay to mild muscle hypotonia and normal intelligence. This is in accordance with the results of Balci et al. [13], who first described white matter abnormalities in patients with *PTEN* gene mutations and otherwise normal development. In contrast, Frazier et al. [31] compared patients with germline heterozygous *PTEN* mutations and autism spectrum disorder (*PTEN*-ASD) with idiopathic ASD patients and healthy controls, and found that prominent white matter and cognitive abnormalities were specifically associated with *PTEN*-ASD patients. White matter abnormalities mediated the relationship between *PTEN* protein reductions and reduced cognitive ability. Intellectual disability was described as a possible clinical sign of patients with the *PTEN* gene mutation. In our cohort, we found a broad range of intellectual abilities. The majority of our patients demonstrated a mean IQ within the normal range.

This is in accordance with others [30] who tested cognitive function in selected individuals with *PTEN* mutations and reported that mean IQ was average. A limitation of our retrospective study is that we did not perform a standardized neurodevelopmental evaluation in our patients. Not all patients received IQ testing, sometimes because parents or referring physicians had no evidence for intellectual disabilities. Subjects in whom cognitive testing was performed, were tested at their respective local institutions by different physicians. We were thus unable to give precise information on partial disturbances of performance in this study cohort. Autism spectrum disorder (ASD) or autism spectrum disorder-features were only tested in a minority of our patients, even though we cannot exclude that more patients might have ASD or at least ASD-features, as the prevalence of ASD in PHTS is estimated around 22% [8,29]. A delay in motor development and motor coordination were observed frequently, which could be reflected by the delayed ability of independent walking as a milestone of motor development. Another limitation is that the cMRIs did not follow a uniform examination protocol as they were part of the local clinical work up of macrocephaly. It would be favorable if all children could have been examined at determined ages with a more comparable technical quality.

Coming back to EPVS and white matter abnormalities once more, we agree that EPVS and white matter abnormalities are part of the clinical spectrum of patients with *PTEN* mutations and do not need further clarifications, if the diagnosis of PHTS has already been made [28]. Even though all MRI findings of our cohort have been described previously, we can underline the high prevalence of EPVS and white matter abnormalities in a relatively large group of pediatric PHTS patients. Since, in our pediatric cohort, EPVS (95%) and white matter abnormalities (37%) seem to have a higher or at least equal prevalence to penile freckling (56%), lipoma (56%) and hemangioma (18.5%), we want to emphasize these attributes as new diagnostic features of PHTS in childhood.

In our study cohort, the combination of macrocephaly and EPVS could help to establish the diagnosis of *PTEN* gene mutation in two cases. But if the association of EPVS and white matter abnormalities were more established and better known as a diagnostic feature of *PTEN* Hamartoma Tumor Syndrome by pediatricians, pediatric neurologists and radiologists, we are convinced that *PTEN* gene analysis would be performed earlier and may establish an earlier diagnosis in many cases.

The criteria in Table 2a,b might thus serve as a tool for indicating a molecular diagnostic workup to detect *PTEN* gene mutation in children and adolescents.

## 5. Conclusions

EPVS and white matter abnormalities occur at a higher or at least comparably high prevalence compared to penile freckling, lipoma or hemangioma in this pediatric cohort and should therefore be considered as an important diagnostic feature of PHTS in childhood. Most pediatric patients demonstrated a delay in motor development, but processed a normal intelligence, even though intellectual abilities were very variable.

## Figures and Tables

**Table 1:** Cerebral MRI scan and neurological features in 27 pediatric PHTS subjects.

Pat.No	Sex	Mutation/Deletion in *PTEN* Gene (Localisation)	Age at DIAGNOSIS (Years)	Age at cMRI (Years)	Results of cMRI Scan	IQ > 85	Delay in Motor Develop-ment	Muscle Hypotonia	Confirmed Autism	More Detailed Description of Neurological Features and Academic Performance	Age at Start of Walking Independently (Months)
1	male	c.389G > A; Arg130 Gln (exon 5)	3.8	n.d.	n.d.	+	+	-	-	moderate delay in motor development, normal intelligence, secondary school −>university	22
2	male	c.389G > A; Arg130 Gln (exon 5)	4	0.25 2.5	enlarged perivascular spaces	-	+	+	+	muscle hypotonia, autism, developmental delay in motor and language development, no expressive speech	26
3	male	c.540C > A; p.Y180X (exon 6)	5.3	6	enlarged perivascular spaces	+	+	-	-	delay of fine motor skills, normal intelligence, secondary school	18
4	male	c.737C > T.p.Pro246Leu (exon 7)	1.3		normal MRI scan (reported)	+	-	-	-	None, normal intelligence, secondary school	17
5	male	c.209 + 5G > A (Intron 3)	2	1.75	white matter abnormalities, (periventricular posterior white matter), enlarged perivascular spaces	+	+	-	-	delay of gross and fine motor skills, normal intelligence, impulsivity, secondary school	18
6	male	c.445C > T; Gln149X (exon 5)	3	2.0	white matter abnormalities (posterior horn up to parietal white matter; smaller frontal and periventricular lesions), enlarged perivascular spaces	+	-	-	-	None, normal intelligence, elementary school	18
7	male	c.509G > A; pSer170Asn (exon 6)	4	n.d.	n.d.	+	+	-	-	moderat delay in fine motor skills, normal intelligence, elementary school	14.5
8	male	heterozygous deletion (exon 1–2)	8	4.5 4.75 5.3	Ventriculo-peritoneal shunt, enlarged perivascular spaces	-	+	-	(-)	social behaviour problems, impulsivity, developmental delay, pseudotumor cerebri, Difficulties in regular school	?
9	male	partial deletion (exon 6)	1.5	1 2.5 4.5	Periventricular, occipital, parietal and and smaller frontal white matter abnormalities; enlarged perivascular spaces	+	+	+	-	muscle hypotonia, moderate delay in motor development, normal intelligence, secondary school diagnosis of PHTS because of MC and EPVS	20
10	male	c.697C > T;pArg233*(exon 7)	11	n.d.	n.d.	+	-	-	-	None, normal intelligence, secondary school	18
11	male	c.959T > G (p.Leu320*)	7.5	0.3 7.8 8.3 9.3	Cavernoma right side cerebellum, enlarged perivascular spaces; slight parieto-occipital white matter abnormalities	+ IQ 91	-	+	-	muscle hypotonia, difficulties in logical reasoning, impulsivity normal intelligence: HAWIK IV with 8 years: IQ 91 Special needs school	14
12	male	c.987dup T (p.Lys330*) (exon 8)	6.5	n.d.	n.d.	+ IQ 84	+	+	-	muscle hypotonia WPPSI-III, 2009–HAWIVA-III with 6 years: IQ 84, elementary school	24
13	male	c.(492 + 1_493–1)_(1026 + 1_1027–1)del	0.9	0.6	enlarged perivascular spaces	+	+	+	-	muscle hypotonia, delay in fine motor skills, normal intelligence, kindergarten	19
14	male	heterozygous deletion *PTEN* and *BMPR1A* Gene	0.7	0.75 2	arachnoid cysts left and right of the pineal region, enlarged perivascular spaces, parietal and temporal white matter abnormalities (left sided pronounced), parietal Pacchioni granulation	+	-	+	-	muscle hypotonia	18
15	male	c.800_801delAG (exon 7)	10	4.5	enlarged perivascular spaces, slight parietal white matter abnormalities	−/+ IQ with 4.5 years: 70; with 5.9 years: IQ 89	+	-	-	developmental delay in speech, cognition and motor development, HAWIWA III with 4.5 years: 70; HAWIVA III with 5.9 years: IQ 89, special needs school	14
16	male	c.464a > G; p.Tyr155Cys	12	11.75	enlarged perivascular spaces	+ IQ 85	+	-	-	problems in sense of balance, dyslexia, panic attacks, diagnosis of PHTS because of MC and EPVS	?
17	male	p.Arg130Ter*;c.388C > T	4.5	n.d.	n.d.	+	+	-	-	delay in motor development, normal intelligence	30
18	male	c.266C > G (p.Pro89Arg)	9	0.75 2.25 8	subependymal heterotopia at the top of the right lateral ventricle, enlarged perivascular spaces	+ IQ 93	+	+	+	delay in language and motor development. autism, ADHD, muscle hypotonia, obsessive-compulsive disorder, social behaviour problems, HAWIK: IQ 93, special needs school	30
19	female	c.741dupA; p.Pro248Thrfs*5 (exon 7)	13.5	n.d.	n.d.	+	-	-	-	None, secondary school −> university	13
20	female	c.302T > C; p.Ile101Thr (exon 5)	5	n.d.	n.d.	+ IQ 89	+	+	-	global developmental delay, muscle hypotonia, IQ testing: 89	24
21	female	c.762dupA; p.Val255Serfs*43 (exon 7)	5.5	6.2	enlarged perivascular spaces	+ IQ 96	+	++	-	severe muscle hypotonia, difficulties in logical reasoning, HAWIK-IV/WISC-IV: IQ 96, special needs school	24
22	female	c.49C > T;p.Gln17* (exon1)	6.8	8.5	normal MRI scan, but enlarged perivascular spaces (reported)	+ IQ 95	+	+	-	ADHS, orofacial hypotonia, delay in motor development, normal intelligence, IQ with 6 years:95 Problems in elementary school	18
23	female	c.1008C > G;p.Tyr336* (exon 8)	5.8	2.75 3 10.75	extremely large perivascular spaces, arachnoidal cysts	-	+	-	-	problems in sense of balance, ataxia, global developmental delay, special needs school	19
24	female	c.492delG; p.Gly165Glufs*2 (exon 5)	2.8	1.25	normal MRI scan enlarged perivascular spaces	+	-	-	-	None, normal intelligence, secondary school	17
25	female	c.1133_1136del.pArg378ilefs*37 (exon 9)	3.5	1	Chiari malformation type I, enlarged perivascular spaces	?	+	-	-	delay in cross motor skills, language developmental delay, kindergarten	29
26	female	c.389G > A; p.(Arg130 Gln) (exon 5)	2.3	0.8	Supraventricular white matter abnormalities, left-sided; enlarged perivascular spaces	?	+	+	-	muscle hypotonia, delay in language and motor development, kindergarten	28
27	female	c.406T > C(p.Cys136Arg)	3	n.d.	n.d.	?	+	+	-	autism, muscle hypotonia, delay in language development, kindergarten	20

**Table 2:** Tool for indicating a molecular diagnostic workup to detect *PTEN* gene mutation in children and adolescents. (**a**) Major- and minor criteria for indicating molecular testing of the *PTEN* gene mutation in children and adolescents (modified after [19,32]). (**b**) (modified after Tan et al. [8]): Clinical criteria for molecular testing of the *PTEN* gene.

**(a)**			
**Major Criteria**		**Minor Criteria**	
Macrocephaly		Autism spectrum disorder	
Positive family history		Mental retardation (i.e., IQ of 75 and below)	
Facial trichilemmomas (>/= 3)		Esophageal acanthosis	
Oral papilloma		Lipoma	
Macular pigmentation of glans penis		Renal cell carcinoma	
Multiple GI hamartomas or ganglioneuroma		Testicular lipomatosis	
Thyroid carcinoma/adenoma		Other thyroid lesions (e.g., adenoma, multinodular goiter)	
Breast cancer		Vascular anomalies	
Endometrial cancer		Enlarged perivascular spaces in cMRI	
		White matter abnormalities	
**(b)**			
**Analysis of *PTEN* Gene, If**	**Macrocephaly Plus**	**No Macrocephaly/Positive Family History**	**Positive Family History (Positive *PTEN* Gene Mutation)**
	At least one of the following criteria:	2 major criteria	Genetical testing without any other criteria, if a parent is positive for a *PTEN* gene mutation
	autism spectrum disorder or developmental delay	1 major criteria +2 minor criteria	
	dermatologic features, including lipomas, trichilemmomas, oral papillomas, penile freckling	3 minor criteria	
	vascular pathologies		
	multiple GI hamartomas or ganglioneuroma		
	thyroid lesions (especially adenoma and carcinoma)		
	enlarged perivascular spaces in cMRI		
